# Communication Skills, Problem-Solving Ability, Understanding of Patients’ Conditions, and Nurse’s Perception of Professionalism among Clinical Nurses: A Structural Equation Model Analysis

**DOI:** 10.3390/ijerph17134896

**Published:** 2020-07-07

**Authors:** Ae Young Kim, In Ok Sim

**Affiliations:** Red Cross College of Nursing, Chung- Ang University, Seoul 06974, Korea; springbreez@naver.com

**Keywords:** communication, clinical nurse, perception of professionalism, patient’s condition, structural equation model

## Abstract

This study was intended to confirm the structural relationship between clinical nurse communication skills, problem-solving ability, understanding of patients’ conditions, and nurse’s perception of professionalism. Due to changes in the healthcare environment, it is becoming difficult to meet the needs of patients, and it is becoming very important to improve the ability to perform professional nursing jobs to meet expectations. In this study method, structural model analysis was applied to identify factors influencing the perception of professionalism in nurses. The subjects of this study were 171 nurses working at general hospitals in city of Se, Ga, and Geu. Data analysis included frequency analysis, identification factor analysis, reliability analysis, measurement model analysis, model fit, and intervention effects. In the results of the study, nurse’s perception of professionalism was influenced by factors of communication skills and understanding of the patient’s condition, but not by their ability to solve problems. Understanding of patient’s condition had a mediating effect on communication skills and nursing awareness. Communication skills and understanding of the patient’s condition greatly influenced the nurse’s perception of professionalism. To improve the professionalism of clinical nurses, nursing managers need to emphasize communication skills and understanding of the patient’s condition. The purpose of this study was to provide a rationale for developing a program to improve job skills by strengthening the awareness of professional positions of clinical nurses to develop nursing quality of community.

## 1. Introduction

Changes in the environment related to climate and pollution are causing health problems and various diseases such as respiratory and circulatory problems, metabolic disorders, and chronic diseases. Moreover, access to modern healthcare facilities has created greater expectations among patients receiving personalized healthcare and high-quality healthcare. As the difficulty of satisfying the demands of patients increases, enhancing nursing capabilities has become increasingly important [1]. To improve this, hospitals are making efforts to change the internal and external environments so as to increase the number of nurses, reduce the length of hospital stays, and enable efficient nursing practice. Despite these efforts, the workloads of nurses and the demand for clinical nurses are continuously increasing [2,3]. As a result, nurses are developing negative attitudes and prejudices toward patients, as well as negative perceptions of professionalism. To address this, the cultivation and strengthening of nursing professionals’ capabilities is essential. 

Nurses’ perception of professionalism is an important element influencing their ability to perform independent nursing, and a good perception of their profession results in a positive approach to solving patients’ problems [4,5]. In addition, the characteristics and abilities of individual nurses can influence the level of care and enable them to understand patients, solve problems, and provide holistic care, which is the ultimate goal of the nursing process [6,7]. Thus, patients expect nurses to not only have medical knowledge of the disease but to also be able to comprehensively assess the patient’s problems and be independent and creative in nursing [8]. This attitude can have a major impact on the quality of nursing services and can inspire pride in the nursing occupation and professional achievement. These findings can also be used by nurses to prevent burnout and maintain professionalism [9,10]. 

To respond to the increasing demands for diverse qualitative and quantitative nursing services and to strengthen the capabilities of nursing professionals, efforts have been made to move nursing education toward scientific and creative education. However, in point-of-care environments, not only are nurses prevented from making independent decisions regarding nursing, but also the diverse personal capabilities necessary for such independent behavior are not sufficiently developed [11]. Therefore, it is important to enhance clinical nurses’ perceptions of the nursing profession; maintain a balance of nursing capabilities; provide novel, high-quality nursing services; and identify assistive nursing education methods and obstructive environmental factors [10]. 

Communication skills involve a person’s ability to accurately understand (through both verbal and non-verbal indications) another person, and sufficiently deliver what the person desires [12,13]. Good communication skills are a primary requirement for providing professional nursing services because they enable an in-depth understanding of patients, solving of complicated problems, and reasonable and logical analysis of situations [14,15,16]. When effective communication takes place, nurses’ problem-solving abilities and perceived professionalism strengthen [17,18]. 

According to Park [19], nurses have difficulties in interpersonal relationships when social tension and interaction skills are low and communication is poor. In addition, these factors are negatively affected not only in the work of the nurse but also in the perception of the profession. Communication skills are associated with both the formation of relationships with patients and the ability to perform holistic nursing [20]. In order to improve and develop the overall nursing function of a clinical nurse like this, it is important to complement the relevant integrated nursing abilities [21,22].

Previous studies have investigated the importance of communication skills for nurses, and the relationships between nurses’ problem-solving ability and their understanding of the patients’ conditions. Nonetheless, data that can comprehensively explain the structural relationships between these qualities and how they affect the job perception of nurses remains insufficient.

Therefore, the present study aims to identify the structural model for the relationships between nurses’ communication skills, problem-solving ability, understanding of patients’ conditions, and nurse’s perception of professionalism. Additionally, the study provides basic data necessary for developing programs for improving nursing abilities.

The purpose of this study is to construct a theoretical model that explains the structural relationships among nurses’ communication skills, problem-solving ability, understanding of patients’ conditions, and nurse’s perception of professionalism. In addition, the study aimed to verify this model using empirical data.

## 2. Materials and Methods 

### 2.1. Study Design

To create and analyze the structural model for clinical nurses’ communication skills, problem-solving ability, understanding of patients’ conditions, and nurse’s perception of professionalism, the theoretical relationships among the variables were developed based on related theories.

**Figure 1:**
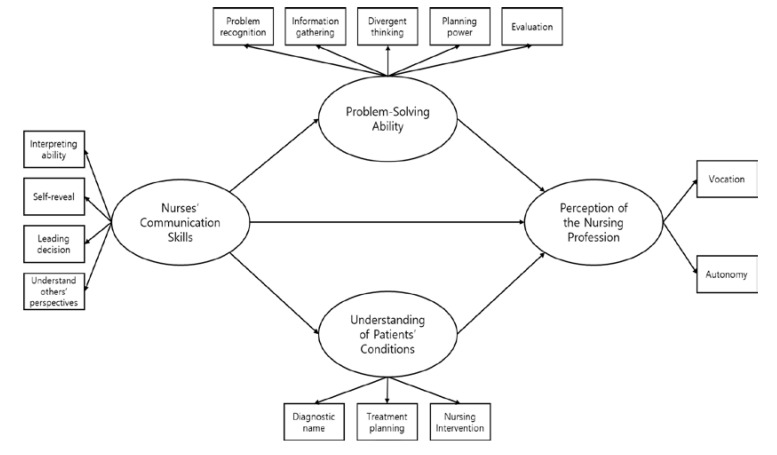
Study model.

In this study, communication skills were set as the exogenous variables, whereas problem-solving ability, understanding of patients’ conditions, and perception of the nursing occupation were set as the endogenous variables. In addition, communication skills were set as the independent variables and nursing job perceptions as the dependent variable. This is because the ability of communication helps to maintain an intimate relationship with the patient and to assess the patient’s condition through each other’s relationship and to solve problems and develop correct understanding. Communication skills, problem-solving ability, and understanding of patients’ conditions were set as the parameters for determining causality. The research model is shown in Figure 1.

### 2.2. Study Participants

The structural equation model has less than 12 measurement variables. The sample size usually requires 200 to 400 participants [23]. A total of 250 participants were selected for the study. In line with ethical standards and practices, participants received a full explanation on the purpose of the study. They were briefed that the information collected would be used for research purposes only. Furthermore, they were informed that they could withdraw from the study at any time. 

### 2.3. Data Collection Method

Data collection for this study was performed by two researchers unrelated to the hospital from April 20 to May 1, 2019. A questionnaire was used to collect data from clinical nurses working in five hospitals in Seoul, Gyeonggi, and Gangwon provinces. Of the 250 questionnaires disseminated, we received 225 completed returns. However, 54 were considered inaccurate, inconsistent, or unsatisfactory for coding purposes. Thus, 171 fully completed valid questionnaires comprised the final dataset for analysis. 

### 2.4. Research Instruments 

#### 2.4.1. Communication Skills 

In this study, the communication skill instrument developed by Lee and Jang [24] was used. Its contents were modified and supplemented to clearly understand the communication skills of nurses. Our questionnaire comprised 20 questions with five questions each concerning “interpretation ability,” “self-reveal,” “leading communication,” and “understanding others’ perspectives.” The answers were rated on a five-point Likert scale ranging from 0 = “strongly disagree” to 4 = “strongly agree.” For this study, the Cronbach’s alpha value was 0.81.

#### 2.4.2. Problem-Solving Ability

The tool developed by Lee [25] was used to measure the problem-solving ability of clinical nurses. The survey comprised 25 questions, with five questions each concerning “problem recognition,” “information-gathering,” “divergent thinking,” “planning power,” and “evaluation.” Items were scored on a five-point Likert scale ranging from 0 = “strongly disagree” to 4 = “strongly agree.” The internal consistency confidence value Cronbach’s alpha was 0.79.

#### 2.4.3. Understanding Patients’ Condition

To measure nurses’ understanding of patients’ conditions, we developed 10 questions by revising and supplementing items from an existing understanding-measurement tool [26]. With a total of ten questions, we measured “diagnostic name,” “patient-treatment planning,” and “nursing intervention processes.” Items were scored using a five-point Likert scale ranging from 0 = “strongly disagree” to 4 = “strongly agree.” The internal consistency confidence value Cronbach’s alpha was 0.81.

#### 2.4.4. Nurse’s Perception of Professionalism 

Nurse’s perception of professionalism was measured using a tool developed by revising the 25 questions created by Kang et al. [1]. With a total of ten questions, we measured “vocation” and “autonomy.” Items were scored using a five-point Likert scale. The internal consistency confidence value Cronbach’s alpha was 0.81.

### 2.5. Data Analysis 

To identify the relationships among the set variables, the data were computed statistically using the program included in IBM SPSS 24.0 and AMOS 23.0. (IBM Corp., Armonk, NY, USA). The analysis methods were as follows:

1. Frequency analysis was conducted to identify the subjects’ demographic and general characteristics.
2. The reliability of the questionnaire was verified using Cronbach’s α coefficients.
3. Confirmatory factor analysis (CFA) was performed to verify the convergent validity of the selected measurement tool.
4. The normality of the data was determined through analyzing the skewness and kurtosis of the measurement variables.
5. The fitness of the model was verified using structural equation modeling (SEM).
6. Bootstrapping was utilized to verify the mediating effect in the set study model, as well as the mediating effects of the nurses’ problem-solving ability and understanding of patients’ conditions.

## 3. Results

### 3.1. Demographic Characteristics

The demographic and general characteristics of the study subjects are shown in Table 1. Overall, 71 respondents were aged 25–29 years (41.5%), representing the most numerous age group. University graduates comprised 113 (66.1%) of the sample, while 50 (29.2%) held graduate degrees, with eight (4.7%) holding master’s degrees. Fifty-three respondents (31.0%) had over seven years of clinical experience, 43 (25.1%) had two to three years of experience, 42 (24.6%) had four to six years of experience, and 33 (19.3%) had less than two years of experience. Additionally, 121 respondents (70.8%) worked at secondary hospitals, while 50 (29.2%) worked at tertiary hospitals; 159 respondents (93.0%) reported that they were general nurses. 

### 3.2. Technical Metrics of the Measurement Variables

The multivariate normality of the findings related to the factors of the latent variables was verified through standard deviations, skewness, and kurtosis. The present study meets the criteria for the skewness and kurtosis values mentioned by Hu and Bentler [27]. 

All sub-factors of the latent variables secured normality. 

In this study, a normal distribution was obtained for each of the four sub-factors of communication skills, five sub-factors of problem-solving ability, three sub-factors for understanding the patient’s condition, and two sub-factors of the nurse’s perception of professionalism as shown in Table 2.

### 3.3. Correlations between the Measured Variables 

The correlations between the measurement variables were analyzed using Pearson’s product–moment correlation coefficient analysis (Table 3). The correlations among all individual measurement variables were found to show a positive correlation.

### 3.4. Confirmatory Factor Analysis of the Measurement Model

This study examined how well the measurement variables represented the latent variables in the measurement model. Each set path coefficient was evaluated using non-standardization factors, standardization factors, and standard errors. The path coefficients refer to the factor loadings in CFA. The standardization factors of the individual paths were shown to be at least 0.50 (except for vocation: 0.36), and the critical ratio (CR) was at least 1.96. This indicated that the measurement tool had good convergent validity (Table 4).

### 3.5. Verification of the Structural Model

**Figure 2:**
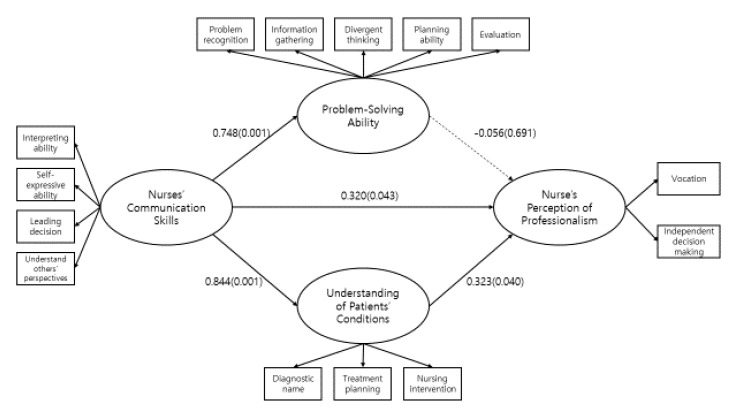
Final model. * χ^2^ = 124.074 (df = 61, *p*<0.001), GFI(Goodness of Fit Index)= 0.90, RMSEA(Root Mean Square Error Approximation)=0.07, NFI(Normed Fit Index)=0.87, IFI(Incremental Fit Index)= 0.93, TLI(Tucker-Lewis Index)= 0.91, CFI(Comparative Fit Index)= 0.92.

The structural model for relationships among clinical nurses’ communication skills, problem-solving ability, understanding of patients’ condition, and nurse’s perception of professionalism that would be suitable for predicting the influencing relationships was verified. Since the fitness index of the modified model was shown to be higher than that of the initial model, the final model for this study was set as shown in Figure 2.

### 3.6. Influencing Relationships between Variables of the Study Model 

The standardization factors and CR values of the final model were examined to determine whether there were direct relationships between communication skills, problem-solving ability, understanding of patients’ conditions, and nurse’s perception of professionalism. The results are shown 

For the relationship between communication ski in Table 5.lls and problem-solving ability, the standardization factor was 0.85 and the CR value was 7.37; communication skills showed a statistically significant effect. Consequently. The relationship between communication skills and understanding of patients’ conditions also showed a statistically significant effect. Consequently, Hypothesis 1 was supported.

For the relationship between communication skills and nurse’s perception of professionalism, the standardization factor was 0.54, and the CR value was 2.02. Communication skills showed a statistically significant effect. Consequently. For the relationship between problem-solving ability and nurse’s perception of professionalism, the standardization factor was −0.056, and the CR value was −0.39. Problem-solving ability had no statistically significant effect. Consequently.

The relationship between nurses’ understanding of patients’ conditions and nurse’s perception of professionalism had a statistically significant effect. Consequently Figure 2 shows the influencing relationships between the study variables of the final study model, considering non-standardization and standardization factors of the relationships between the study variables.

### 3.7. Direct and Indirect Effects of the Variables

To grasp the significance of the mediating effect in the final study model, the direct and indirect effects of each variable were examined. To examine the mediating effect of the problem-solving ability and understanding of patients’ conditions variables, the bootstrapping method provided by the AMOS 23.0 program included in IBM was utilized. The results are shown in Table 6.

The indirect effect of communication skills on nurse’s perception of professionalism through nurses’ understanding of patients’ conditions was statistically significant. That is, clinical nurses’ communication skills have an indirect positive effect on their nurse’s perception of professionalism, with nurses’ understanding of patients’ conditions acting as a parameter. We also found that the effect of communication skills on nurse’s perception of professionalism was statistically significant. Therefore, communication skills have a partially mediated effect on nurse’s perception of professionalism, with understanding of patients’ conditions acting as a parameter. However, communication skills were found to have no indirect positive effect on nurse’s perception of professionalism when problem-solving ability was set as a parameter. 

## 4. Discussion

In this study, we developed and analyzed a hypothetical model regarding clinical nurses’ communication skills, problem-solving ability, and understanding of patients’ conditions, and how these factors influence their nurse’s perception of professionalism.

### 4.1. Effect of Communication Skills on Nurses’ Perception of Professionalism 

Communication skills were found to have statistically significant effects on their relationship with nurses’ problem-solving ability, understanding of patients’ conditions, and nurse’s perception of professionalism. Nurses’ communication skills not only affected their problem-solving ability but also their understanding of patients’ conditions and nurse’s perception of professionalism. Good communication among nurses can reduce uncomfortable situations and improve interactions with patients, which can consequently enhance problem-solving [28]. Supporting our findings, Ancel [17] reported that communication skills afford the maintenance of amicable cooperative relationships with patients across diverse medical classes, thereby enhancing the efficiency of nursing-related problem-solving. 

Nurses’ communication is also closely related to their understanding of patients’ conditions, particularly regarding the treatment processes. Nurses frequently experience difficulties as a result of poor communication with not only patients and their family members but also other medical personnel. Further, poor delivery of explanations and questions affects nurses’ understanding of patients’ situations and problems, and patients can also feel concern regarding whether nurses accurately understand their problems [29]. Nurses frequently experience psychological abuse when communicating with patients and develop stress or discomfort [30]; this can lead to distrustful relationships with and inhibited understanding of patients [31,32]. Vermeir et al. [18] reported that scientific approaches are required to understand patients in-depth. To accurately understand both oneself and others, the most important method is successful communication. Such findings support the present study’s indication that nurses’ communication is a basic means of solving nursing problems, with both actions being interrelated. 

Our finding that nurses’ communication skills are structurally related to their nurse’s perception of professionalism supports the findings of many previous studies. Regarding nurse’s perception of professionalism, Adams et al. [33] as well as Lee and Kim [34] explained that a good perception leads to higher-level capabilities, fostering high-level nursing of patients and the development of autonomous vocation. The above studies reported that, since nurses’ communication skills are related to their nurse’s perception of professionalism, communication skills should be considered a predictor of success. Further, McGlynn et al. [35] recommended positively reinforcing communication skills to improve nurse’s perception of professionalism. This supports the findings of the present study, indicating that communication and nursing professional perception are interrelated.

Thus, communication skills are important for nursing patients. They enable nurses to accurately understand patients’ problems, serve (by forming patient trust) an important function in the process of administering nursing interventions, and positively affect nurses’ perception of their profession. As such, each concept is important. However, nurses working in the clinic are critically aware of their professionalism. In order to reinforce this, communication skills are required, and the emphasis is placed on strengthening the nurses’ ability to solve problems as well as assess and understand patients. As a result, communication skills play an important role in helping nurses understand patients’ problems accurately, build patient trust in nursing interventions, and create structural relationships that have a positive impact on the perception of nursing occupations. Therefore, efforts to improve nurses’ communication skills not only improve their problem-solving abilities and understanding of patients’ conditions but also improve their nurse’s perception of professionalism. To maintain the professionalism of nurses, “competency development programs” would be helpful, thereby emphasizing the need for their application in nursing colleges and clinical practice.

### 4.2. Relationship between Nurses’ Problem-Solving Ability and Nurse’s Perception of Professionalism 

We found clinical nurses’ problem-solving ability to have no positive effect on their perception of professionalism. This contrasts with previous studies, which reported that problem-solving ability is helpful for such perception of professionalism [36]. We also found that problem-solving ability does not affect nursing professional perception through a mediating effect. 

The present findings indicate that the distinctiveness of the fields of nursing should not be overlooked. In nursing organizations that have a culture of discouraging diversity, when negative results are obtained from attempts to solve nursing problems, confusion regarding the identity of nursing professionals means perception of the profession is not reinforced; in many cases, the opposite perception is formed. Furthermore, for those in lower-level positions, nurse’s perception of professionalism is thought to be low because they cannot voice their opinions and have difficulties such as excessive workloads. Although few previous studies have directly examined this, Vermeir et al. [18] explained that, as the role expectation for nurses increases, factors for job turnover increase as a result of a sense of confusion regarding the nurses’ role and increases in stress. These findings indicate that factors that degrade nurses’ problem-solving ability induce skepticism regarding nursing and possibly career change, thereby supporting the findings of this study.

However, in the present study, positive results with low levels of relevancy in the structural model but high correlations were found. It is expected that, if nurses’ environmental conditions are improved and their nursing capabilities are developed so that they can solve nursing problems with confidence, their nursing professional perception will improve.

### 4.3. Relationship between Nurses’ Understanding of Patients’ Conditions and Nurse’s Perception of Professionalism

Our findings indicated that the relationship between nurses’ understanding of patients’ conditions and nurse’s perception of professionalism was statistically significant. This supports Nilsson et al. [21] and Philip et al. [29], who reported that, in the fields of nursing, when patients accurately understand nurses’ instructions or explanations and health information, they can participate in, independently adjust, and engage in creative decision-making related to self-nursing.

McGlynn et al. [35] suggested that understanding patient problems is an important element in resolving negative situations; meanwhile, Heo and Lim [37] indicated that clinical nurses provide high-quality nursing services and develop self-efficacy when they apply professional knowledge and a desire to understand patients’ problems. These study findings accord with our own findings.

The aforementioned findings suggest that the development and application of programs that can enhance nurses’ understanding of patients’ conditions should be emphasized, and that studies of various patient types, the characteristics of patients by age group and hospital areas, as well as the introduction of simulation education programs to improve nurses’ understanding of patients’ conditions should be continuously implemented. 

## 5. Conclusions

This study aimed to verify the structural relationships between clinical nurses’ communication skills and their problem-solving ability, understanding of patients’ conditions, and nurse’s perception of professionalism. We also aimed to identify, through a structural model, the mediating effects of nurses’ problem-solving ability and understanding of patients’ conditions in the relationship between communication skills and nurse’s perception of professionalism.

The findings of this study are as follows (all significance levels = 0.05). In the relationship between communication skills and problem-solving ability, the value of the standardization factor was 0.85 and the CR value was 7.37, indicating that communication skills had a statistically significant effect. In the relationship between nurses’ communication skills and understanding of patients’ conditions, the value of the standardization factor was 0.61 and the CR value was 6.35, indicating that communication skills had a statistically significant effect. In the relationship between communication skills and nurse’s perception of professionalism, the value of the standardization factor was 0.54 and the CR value was 2.02, indicating that communication skills had a statistically significant effect. However, in the relationship between problem-solving ability and nurse’s perception of professionalism, the value of the standardization factor was −0.05 and the CR value was −0.39, indicating that problem-solving ability has no statistically significant effect. Finally, in the relationship between nurses’ understanding of patients’ conditions and nurse’s perception of professionalism, the value of the standardization factor was 0.56, and the CR value was 2.14, indicating that nurses’ understanding of patients’ conditions has a statistically significant effect. 

There are some limitations to this study. First, as we only examined nurses at secondary and tertiary university hospitals, our findings may not be generalizable to all clinical nurses. Replication studies examining a range of levels of medical institutions and associated workers are necessary. Second, the structural relationship between problem-solving ability and the nurse’s perception of professionalism turned out to be insignificant or mediated. Subsequent studies on the various approaches to revisit this structural relationship should be performed. Third, theories should be systematically developed to establish the values of the nursing profession, and additional studies are necessary to explore other variables. 

## Figures and Tables

**Table 1:** Participants’ general characteristics (*N* = 171, %).

Characteristics		Frequency	Rate (%)
Age	<24 years	33	19.3
	25–30	71	41.5
	30–40	39	22.8
	>40	28	16.4
Academic record	College	50	29.2
	University	113	66.1
	Graduate school	8	4.7
Annual income ($ US)	17,000–20,000	31	18.1
	20,000–25,000	22	12.9
	25,000–30,000	33	19.3
	30,000–35,000	38	22.2
	35,000–40,000	28	16.4
	>40,000	19	11.1
Clinical experience	<2 years	33	19.3
	2–3 years	43	25.1
	4–7 years	42	24.6
	>7 years	53	31.0
Affiliated medical institution	Secondary hospital (General hospital, hospital)	121	70.8
	Third hospital (Advanced general hospital)	50	29.2
Position	General nurse	159	93.0
	Charge nurse	7	4.1
	Head nurse	5	2.9
Total		171	100

**Table 2:** Technical metrics of the measurement variables (*N* = 171).

Latent Variable Observed Variable		Mean	Standard Deviation	Skewness	Kurtosis
Nurses’ communication skills	Interpretation ability	3.81	0.51	−0.29	0.85
	Self-reveal	3.45	0.51	−0.09	−0.17
	Leading communication	3.32	0.57	0.02	−0.36
	Understand others’ perspectives	3.59	0.53	−0.22	0.46
Problem-solving ability	Problem recognition	3.71	0.47	0.02	−0.02
	Information-gathering	3.41	0.53	0.17	−0.24
	Divergent thinking	3.23	0.57	0.13	−0.29
	Planning power	3.66	0.49	0.03	1.04
	Evaluation	3.71	0.49	0.24	−0.25
Understanding of patients’ conditions	Diagnostic name	3.76	0.53	−0.00	0.28
	Treatment planning	3.50	0.48	0.07	0.42
	Nursing intervention processes	3.55	0.65	−0.04	0.27
Nurse’s perception of professionalism	Autonomy	3.14	0.67	−0.22	−0.58
	Vocation	3.10	0.62	−0.16	0.54

**Table 3:** Correlations between the observed variables.

	1	2	3	4	5	6	7	8	9	10	11	12	13	14
Nurses’ communication skills														
Interpretation ability	1													
Self-reveal	0.30	1												
Leading communication	0.24	0.60	1											
Understand others’ perspectives	0.52	0.42	0.50	1										
Problem-solving ability														
Problem recognition	0.49	0.30	0.36	0.49	1									
Information gathering	0.19	0.30	0.33	0.44	0.35	1								
Divergent thinking	0.36	0.35	0.34	0.39	0.31	0.49	1							
Planning power	0.30	0.23	0.33	0.34	0.49	0.21	0.30	1						
Evaluation	0.39	0.29	0.23	0.33	0.49	0.38	0.40	0.36	1					
Understanding of patients’ conditions														
Diagnostic name	0.30	0.25	0.35	0.28	0.31	0.21	0.29	0.29	0.37	1				
Treatment planning	0.33	0.31	0.39	0.30	0.36	0.26	0.28	0.25	0.28	0.65	1			
Nursing intervention processes	0.34	0.26	0.41	0.33	0.39	0.22	0.27	0.33	0.37	0.60	0.72	1		
Nurse’s perception of professionalism														
Autonomy	0.12	0.12	0.22	0.29	0.28	0.29	0.25	0.22	0.23	0.25	0.22	0.24	1	
Vocation	0.29	0.42	0.42	0.40	0.36	0.32	0.24	0.22	0.28	0.34	0.39	0.35	0.28	1

**Table 4:** Confirmatory factor analysis of the measurement model.

Directions		+Estimate (*p*)	StandardizAtion Factor (β)	Standard Error	CR
Understand others’ perspectives	← Nurses’ communication skills	1.00	0.73		
Leading communication		1.02	0.69	0.12	8.21 ***
Self-reveal		0.87	0.66	0.11	7.85 ***
Interpretation ability		0.75	0.57	0.11	6.82 ***
Evaluation	← Problem-solving ability	1.00	0.64		
Planning power		0.86	0.56	0.14	6.06 ***
Divergent thinking		1.08	0.59	0.17	6.36 ***
Information gathering		0.98	0.58	0.15	6.23 ***
Problem recognition		1.05	0.71	0.14	7.30 ***
Nursing intervention processes	← Understanding of patients’ conditions	1.00	0.82		
Treatment planning		0.78	0.87	0.06	11.90 ***
Diagnostic name		0.73	0.74	0.07	10.24 ***
Autonomy	← Nurse’s perception of professionalism	1.00	0.79		
Vocation		0.33	0.36	0.09	3.42 ***

*** *p* < 0.001; CR: critical ratio.

**Table 5:** The relationships between the human effects of the measurement model.

Directions			Estimate (*p*)	Standardization Factor (β)	Standard Error	CR
Nurses’ communication skills	→	Problem-solving ability	0.74	0.85	0.10	7.37 ***
Nurses’ communication skills	→	Understanding of patients’ conditions	0.84	0.61	0.13	6.35 ***
Nurses’ communication skills	→	Nurse’s perception of professionalism	0.32	0.54	0.15	2.02 *
Problem-solving ability	→	Nurse’s perception of professionalism	−0.05	−0.11	0.14	−0.39
Understandingof patients’ conditions	→	Nurse’s perception of professionalism	0.32	0.56	0.10	2.14 *

* *p* < 0.05, *** *p* < 0.001; CR: critical ratio.

**Table 6:** Mediating effect analysis.

Directions					Direct Effects	Indirect Effects	Gross Effects
Nurses’ communication skills	→			Understanding of patients’ conditions	0.61 ***	-	0.61 ***
Understanding of patients’ conditions	→			Nurse’s perception of professionalism	0.56 *	-	0.56 *
Nurses’ communication skills	→	Understanding of patients’ conditions	→	Nurse’s perception of professionalism	0.54 *	0.34 *	0.88 *

* *p* < 0.05, *** *p* < 0.001
